# The M3 Muscarinic Receptor Is Required for Optimal Adaptive Immunity to Helminth and Bacterial Infection

**DOI:** 10.1371/journal.ppat.1004636

**Published:** 2015-01-28

**Authors:** Matthew Darby, Corinna Schnoeller, Alykhan Vira, Fiona Culley, Saeeda Bobat, Erin Logan, Frank Kirstein, Jürgen Wess, Adam F. Cunningham, Frank Brombacher, Murray E. Selkirk, William G. C. Horsnell

**Affiliations:** 1 Institute of Infectious Disease and Molecular Medicine, International Centre for Genetic Engineering and Biotechnology and Division of Immunology, University of Cape Town, Cape Town, South Africa; 2 Department of Life Sciences, Sir Ernst Chain Building, South Kensington Campus, Imperial College London, London, United Kingdom; 3 National Heart and Lung Institute, St.Mary’s Campus, Praed Street, Imperial College London, London, United Kingdom; 4 Medical Research Council Centre for Immune Regulation, School of Immunity and Infection, University of Birmingham, Birmingham, United Kingdom; 5 Molecular Signaling Section, Laboratory of Bioorganic Chemistry, National Institute of Diabetes and Digestive and Kidney Diseases, National Institutes of Health, Bethesda, Maryland, United States of America; University of California San Francisco, UNITED STATES

## Abstract

Innate immunity is regulated by cholinergic signalling through nicotinic acetylcholine receptors. We show here that signalling through the M3 muscarinic acetylcholine receptor (M3R) plays an important role in adaptive immunity to both *Nippostrongylus brasiliensis* and *Salmonella enterica* serovar Typhimurium, as M3R^-/-^ mice were impaired in their ability to resolve infection with either pathogen. CD4 T cell activation and cytokine production were reduced in M3R^-/-^ mice. Immunity to secondary infection with *N. brasiliensis* was severely impaired, with reduced cytokine responses in M3R^-/-^ mice accompanied by lower numbers of mucus-producing goblet cells and alternatively activated macrophages in the lungs. Ex vivo lymphocyte stimulation of cells from intact BALB/c mice infected with *N. brasiliensis* and *S. typhimurium* with muscarinic agonists resulted in enhanced production of IL-13 and IFN-γ respectively, which was blocked by an M3R-selective antagonist. Our data therefore indicate that cholinergic signalling via the M3R is essential for optimal Th1 and Th2 adaptive immunity to infection.

## Author Summary

Recent data indicate that acetylcholine (ACh), a neurotransmitter which regulates a variety of physiological functions, also influences the immune system, and that lymphocytes have the capacity to synthesise and release ACh, controlling local innate immune responses and suppressing inflammation. Thus far however there has been little evidence to suggest that ACh influences adaptive immunity, characterised by activation and effector functions of lymphocytes. We show here that during the immune response to two different pathogens, ACh signals through muscarinic receptors, and the M3 receptor subtype specifically, resulting in enhanced activation and cytokine production by ‘helper’ T lymphocytes which protect the host against infection.

## Introduction

The role of acetylcholine (ACh) as a neurotransmitter is well established, both in the central nervous system and the periphery, where it regulates smooth muscle contraction and many other functions of the autonomic nervous system. Cholinergic signalling also influences the immune system, most notably in the cholinergic anti-inflammatory pathway, which results in the α7 nicotinic receptor subunit-dependent inhibition of macrophage TNF-α, IL-1β and IL-6 production [1,2]. The influence of cholinergic signalling on adaptive immunity however is largely unexplored, although there is evidence that nicotinic receptors influence B lymphocyte development and activation [3]. Expression of both nicotinic receptors (nAChRs) and muscarinic receptors (mAChRs) is affected by CD4 T cell activation *in vitro* [4], and mAChRs influence differentiation of CD8 T cells in vivo [5]. To our knowledge, nothing is known about the role of mAChRs in the adaptive response to infection.

*Nippostrongylus brasiliensis* is a common laboratory pathogen used to study T helper 2 immune response-mediated disease resolution, and biologically closely resembles the important human hookworms *Ancylostoma duodenale* and *Necator americanus* [6]. The Th2 response drives resolution of infection, and IL-13 signalling through IL-4Rα is an important component of the protective response [7]. This signalling pathway also enhances smooth muscle contractility, which is thought to contribute to parasite expulsion [8,9]. Previous studies in our laboratory showed delayed parasite expulsion in mice with smooth muscle cells deficient in IL-4Rα. Associated with this defect was reduced Th2 cytokine production, delayed goblet cell hyperplasia and lower expression of the M3 muscarinic receptor (M3R) in the intestine [10]. The mAChR family consists of five subtypes (M1-M5) of G protein-coupled receptors [11], which regulate a range of physiological activities including heart rate, smooth muscle contractility, and endocrine and exocrine gland secretion [12–14]. The M3R is the major mAChR expressed on smooth muscle, and drives contractile responses in the ileum [15]. Our previous investigation determined that upregulation of M3R expression induced by *N. brasiliensis* infection is related to IL-4Rα, sensitive to host immunity, and may therefore also contribute to the immune response [10].

In this study, we investigated the contribution of signalling through the M3R to protective immunity against *N. brasiliensis*, using both infection of M3R gene deficient mice (M3R^−/−^ mice) and *ex vivo* CD4 T cell assays. M3R deficiency significantly abrogated the ability of BALB/c mice to launch an effective adaptive immune response to primary and secondary infection, and underlying this defect were reduced CD4 T cell-associated protective cytokine responses. Stimulation of CD4 T cells from *N. brasiliensis*-infected wild-type (WT) control mice with ACh and muscarinic agonists enhanced their secretion of Th2 cytokines, and this effect could be blocked by muscarinic and M3R-selective antagonists. A similar impairment in immunity was found in M3R^−/−^ mice following systemic infection with *Salmonella typhimurium*. Immunity to *S. typhimurium* is dependent on a robust Th1 immune response [16], with production of IFN-γ by CD4 T cells critical for host protection and bacterial clearance [17]. In the absence of M3R expression, strikingly higher bacterial loads were observed, which again correlated with impaired CD4 T cell cytokine responses. *Ex vivo* stimulation of lymphocytes from *S. typhimurium*-infected WT mice with ACh and muscarinic agonists enhanced IFN-γ secretion, and this effect was blocked with an M3R-selective antagonist. Collectively, these results show for the first time that the magnitude and efficacy of the adaptive response to infection depends on cholinergic signalling via the M3R.

## Results

### M3R expression does not influence immune cell populations or the ability to polarise to Th1 and Th2 subsets in uninfected mice

**Figure 1:**
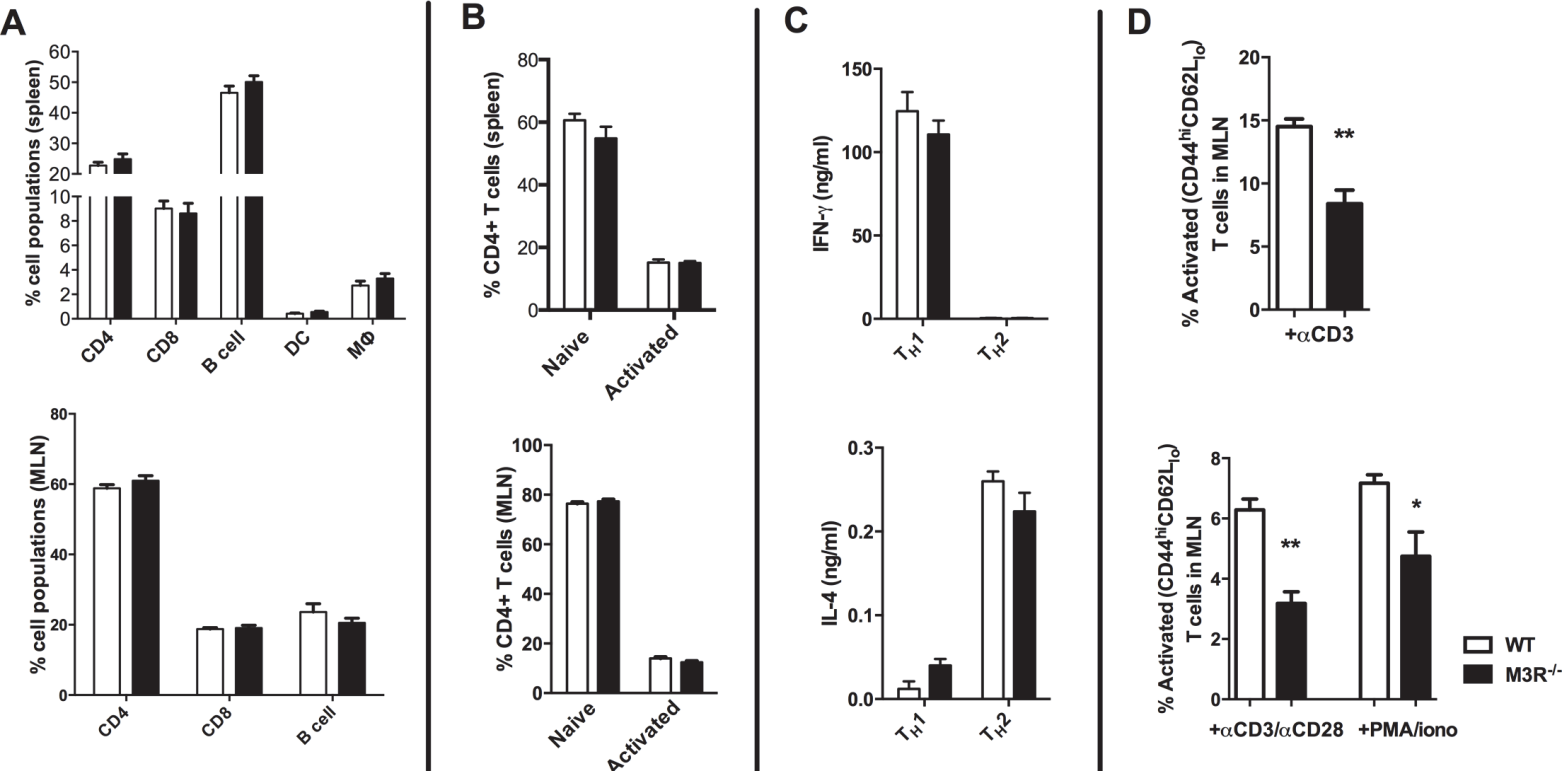
Naïve M3R^−/−^ mice have an equivalent composition of immune cells as WT BALB/c mice but M3R^−/−^ CD4 T cells have an impaired ability to activate. (A) Proportions of splenic immune cell populations (CD4, CD8, B cell, dendritic cell and macrophages) and MLN lymphoid populations (CD4, CD8 and B cells) in naïve mice. (B) Activation status (CD44 and CD62L) of splenic and MLN CD4 T cells in naïve animals. (C) The ability of naïve cells to be polarised ex vivo by priming to Th1 and Th2 phenotypes. (D) Ability of naïve CD4 T cells in whole MLN and CD4 only cell preparations to express activation markers in response to stimulation with anti-CD3 or PMA/ionomycin. White bar: WT BALB/c mice; black bar: M3R^−/−^ mice. Data are shown as mean± SEM, n = 6; Representative of 2 separate experiments.

To determine if expression of the M3R inherently influenced steady-state immune cell populations, we analysed the composition of leukocytes in secondary lymphoid tissues. There was no significant difference in the relative proportions of B cells, CD4 and CD8 T cells, dendritic cells or macrophages in spleens from naïve WT and M3R^−/−^ mice (Fig. 1A), and equivalent proportions of B cells, CD4 and CD8 T cells were also recorded in the mesenteric lymph nodes (MLN) (Fig. 1A). M3R expression also did not appear to affect T cell activation status: WT and M3R^−/−^ mice had equal proportions of naïve (CD3+CD4+CD44^lo^CD62L^hi^) and activated (CD3+CD4+CD44^hi^CD62L^lo^) CD4 T cells in spleen and MLN (Fig. 1B). Furthermore, *in vitro* differentiation of CD4 T cells into Th1 populations by addition of IFN-γ and anti-IL-4 antibody, or Th2 populations via IL-4 and anti-IFN-γ antibody, resulted in equivalent cytokine responses in WT and M3R^−/−^ mice (Fig. 1C). However, anti-CD3 or PMA/ionomycin stimulation of either whole MLN cell suspensions or sorted CD4 T cells for 24 hours demonstrated an impaired ability of M3R−/− CD4+ T cells to express markers of activation when compared to WT CD4+ cells (Fig. 1D). This finding shows that an absence of M3R expression in CD4 T cells results in an impaired ability to respond to a non-specific activating stimulus.

### M3R expression is required for optimal immunity to *N. brasiliensis*

**Figure 2:**
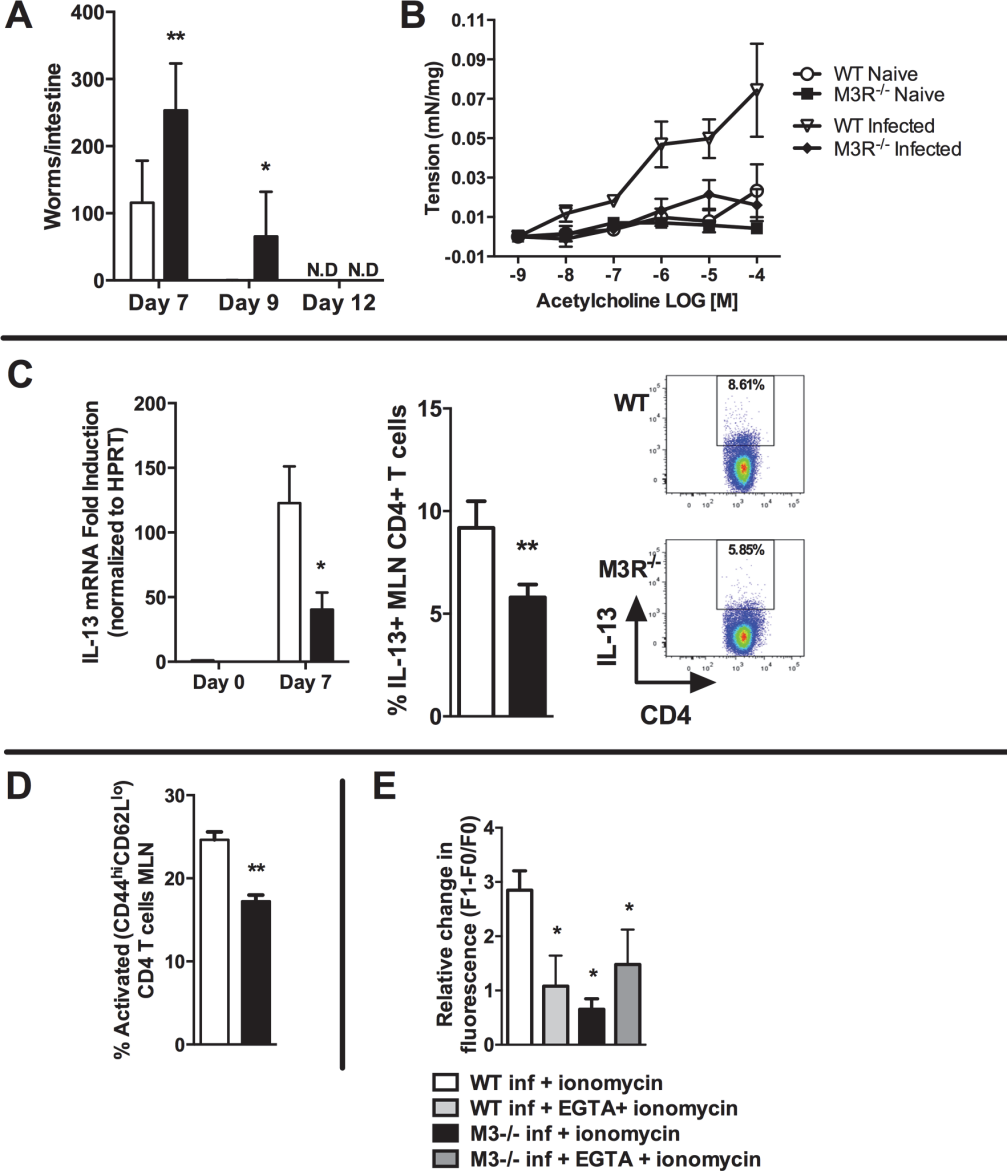
M3R deficient mice exhibit delayed clearance of a primary *N. brasiliensis* infection and impaired T cell-associated protective responses. (A) Adult worm numbers in the small intestine were elevated in M3R^−/−^ mice in comparison to WT BALB/c on day 7 and 9 p.i. (B) Hypercontractile responses of jejunum in response to varying doses of acetylcholine measured at day 9 p.i. were absent in M3R^−/−^ mice. (C) Production of IL-13 by CD4 T cells is impaired in M3R^−/−^ mice: IL-13 mRNA levels and proportion of IL-13^+^ CD4 T cells from the MLN at day 7 p.i. (D) Proportion of activated (CD44^hi^ CD62L^lo^) CD4 T cells in MLN at day 7 p.i. (E) Ca^2+^ mobilisation in response to ionomycin in CD4 T cells in MLN at day 7 p.i. Data are shown as mean ± SEM, n = 4–6 and are representative of at least two independent experiments. Statistical significance was calculated using the Mann-Whitney two-tailed t test and denoted by * p<0.05, ** p<0.01. White bar: WT BALB/c mice; black bar: M3R^−/−^ mice.

We determined whether the M3R contributed to CD4+ T cell immune responses in an *N. brasiliensis* infection. Primary infection with *N. brasiliensis* was self-resolving in WT BALB/c control mice, with parasite clearance from the small intestine by day 9 post-infection (p.i.). M3R^−/−^ mice exhibited delayed expulsion of worms, with significantly higher recovery of parasites from the intestine at days 7 and 9 p.i. (Fig. 2A). M3R^−/−^ mice have previously been shown to be refractory to ACh-induced smooth muscle contraction [15], and intestinal smooth muscle hypercontraction is hypothesised to be a potential effector mechanism for expulsion of nematode parasites [9]. We observed increased ACh-driven smooth muscle contraction compared to naïve animals in infected WT mice but not infected M3R^−/−^ mice (Fig. 2B), suggesting that M3R-mediated smooth muscle hypercontractility may indeed contribute to parasite expulsion. Surprisingly, in addition to this altered physiological response, levels of mRNA for IL-13 were reduced 4-fold in mesenteric lymph node (MLN) cells of M3R^−/−^ mice compared to those of WT controls at day 7 p.i. (Fig. 2C). This was associated with reduced numbers of IL-13^+^ CD4 T cells (Fig. 2C). Additionally, M3R^−/−^ mice had reduced CD3^+^CD4^+^CD44^hi^CD62L^lo^ effector memory (activated) T cells in the MLN when compared to WT mice (Fig. 2D). Elevation of intracellular calcium (Ca^2+^)_i_ is an essential event which is necessary for CD4 T cell activation and cytokine production [18]. We tested whether CD4 T cells from *N. brasiliensis*-infected M3R^−/−^ mice had a reduced ability to mobilise Ca^2+^_i_. This was the case; elevation of Ca^2+^_i_ by the calcium ionophore ionomycin was significantly impaired in M3R^−/−^ CD4 T cells when compared to WT CD4 T cells. No difference was observed between M3R^−/−^ and WT CD4 T cells when extracellular calcium was chelated by EGTA, although the response was much lower, suggesting that lack of the M3R impaired uptake of extracellular calcium across the plasma membrane. These data indicate that M3R expression is required for optimal T cell activation and protective immunity to primary infection by *N. brasiliensis*.

### Cholinergic stimulation of the M3R potentiates cytokine production in CD4 T cells

**Figure 3:**
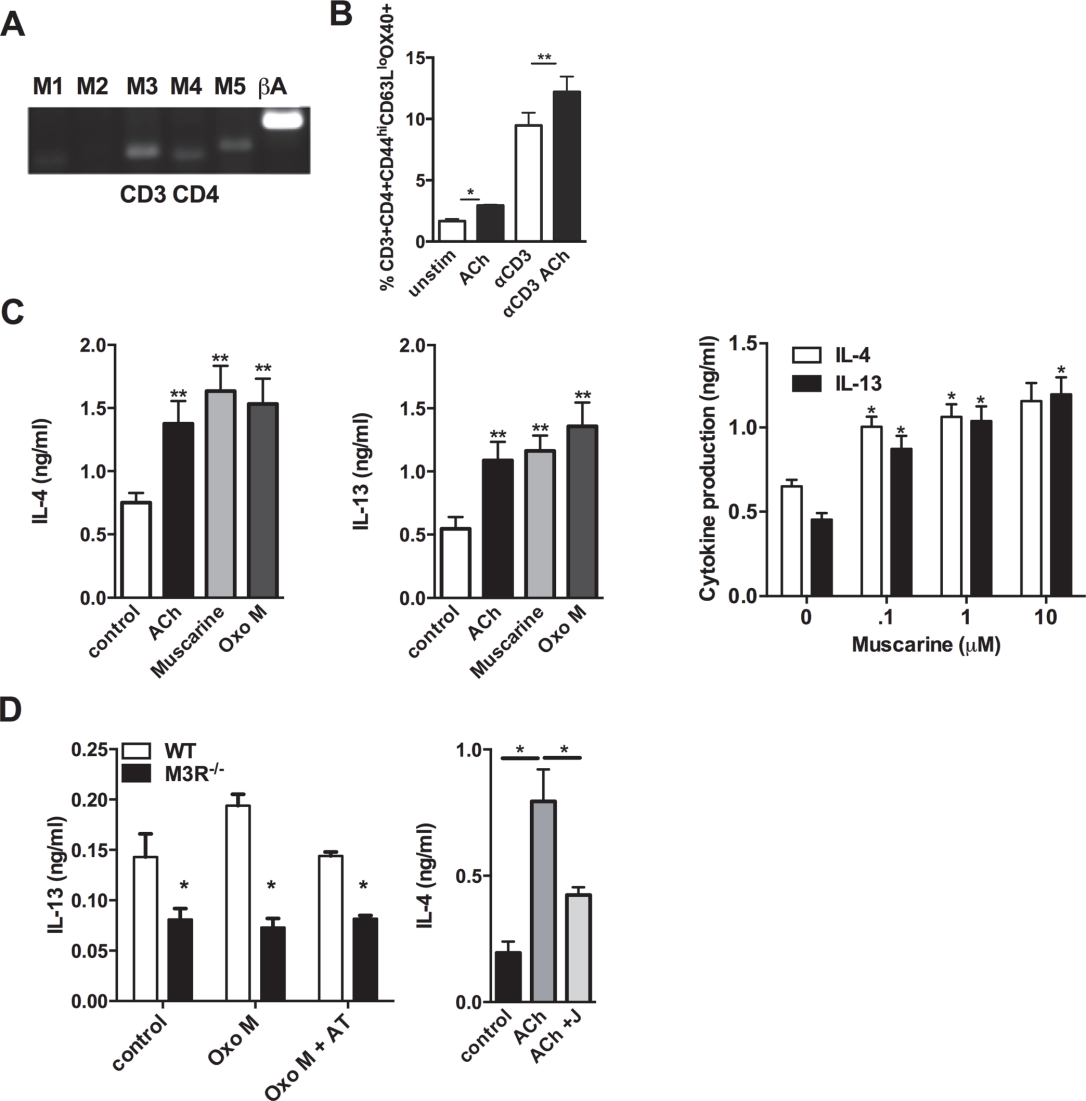
Signalling via the M3R potentiates Th2 cytokine production during *N. brasiliensis* infection. (A) Detection of mRNA for M1-M5 muscarinic receptors and β-actin (βA) in CD4 T cells isolated from MLN. (B) Ox40 expression on effector memory (CD44^hi^CD62L^lo^) subsets of CD4 T cells day 7 p.i. (C) Cytokine secretion by MLN cells day 7 p.i. after stimulation with sub-optimal anti-CD3 and muscarinic receptor agonists (10 μM, 48 hrs) and dose response to muscarine. Asterisks indicate significant differences between control and cholinergic stimulations. (D) IL-13 production from *N. brasiliensis* infected (day 7) WT and M3R^−/−^ MLN cells after sub-optimal anti-CD3 stimulation plus agonists ACh and Oxotremorine M (Oxo M) at 10 μM and the muscarinic receptor antagonist atropine (AT, 100 μM) or the M3R-selective antagonist J104129 (J, 40 nM). Data are shown as mean ±SEM, n = 6 and are representative of at least two independent experiments. *p<0.05, **p<0.01 determined by the Mann-Whitney two-tailed t test.

CD4 T cells isolated from the MLN of WT BALB/c mice 7 days p.i. with *N. brasiliensis* expressed mRNA for the M1, M3, M4 and M5 receptor subtypes (Fig. 3A). Cholinergic stimulation influenced several parameters of CD4 T cell function which are important in the immune response to infection. ACh enhanced expression of Ox40 on effector memory CD4 T cells either directly, or in cells stimulated with sub-maximal concentrations of anti-CD3 (Fig. 3B). When cells were treated with anti-CD3 in the presence or absence of ACh or the muscarinic agonists muscarine and oxotremorine-M (oxo-M) all agonists enhanced production of IL-4 and IL-13 approximately 2-fold, and potentiation of cytokine production by muscarine was demonstrated to be dose-dependent (Fig. 3C). Cholinergic enhancement of IL-13 secretion was blocked by the pan-specific muscarinic receptor antagonist atropine, and no enhancement was observed in cells from M3R^−/−^ mice (Fig. 3D). Further confirmation that the cholinergic co-stimulatory signal acts through the M3R was provided by use of the M3R-selective antagonist J104129, which blocked potentiation of cytokine secretion by ACh (Fig. 3D).

### M3R receptor expression is required for effective recall immunity to *N. brasiliensis*

**Figure 4:**
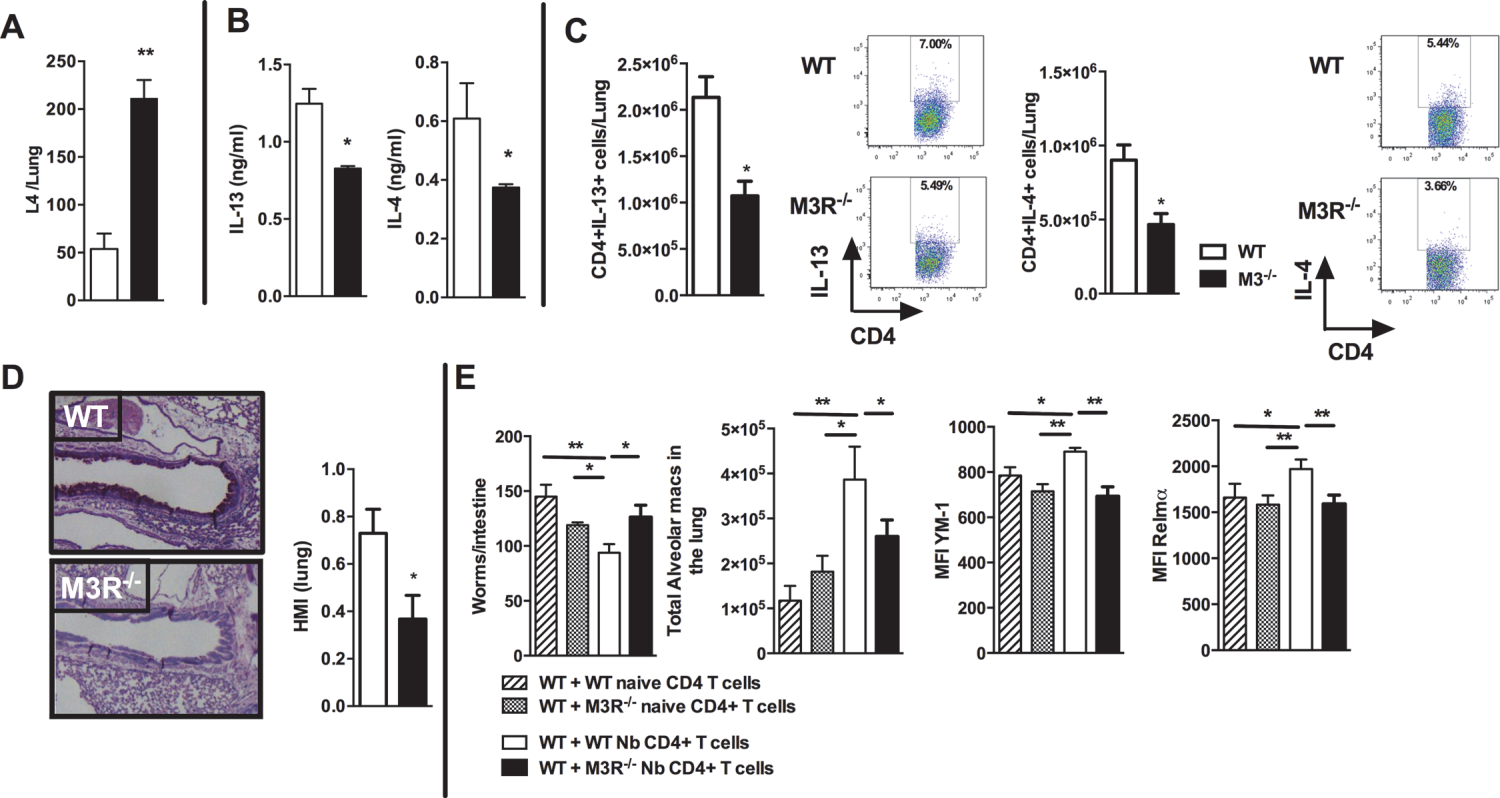
M3R deficient mice have impaired memory immune responses during secondary infection with *N. brasiliensis*. (A) Number of *N. brasiliensis* larvae recovered from the lungs at day 2 post-secondary infection. (B) Levels of IL-13 and IL-4 in total lung homogenates at day 2 p.i. (C) Intracellular IL-13 and IL-4 cytokine production in lung CD4 T cells at day 2 post secondary infection. (D) Goblet cells in the lungs visualised by Periodic Acid Schiff (PAS) staining and enumerated by Histological Mucus Index (HMI) at day 2 p.i. White bar = WT BALB/c mice, black bar = M3R^−/−^ mice. (E) Recovery of adult worms from the intestines of WT BALB/c mice, alveolar macrophage numbers and expression of YM1 and RELMα after adoptive transfer of 5 × 10^5^ CD4 cells from naïve WT mice, WT mice infected with *N. brasiliensis*, naïve M3R^−/−^ mice and infected M3R^−/−^ mice, and infection with *N. brasiliensis* thereafter. Data are shown as mean ± SEM, n = 6 and are representative of two independent experiments. Statistical significance was calculated using the Mann-Whitney two-tailed t test and denoted by *p<0.05, **p<0.01.

To test if cholinergic regulation of CD4 T cell cytokine responses influenced adaptive immunity, we next examined how this affected the outcome of secondary infection, as this requires a CD4 T cell-driven Th2 immune response in the lung [19,20]. Immunity to secondary infection was strikingly abrogated in M3R^−/−^ mice, with higher numbers of larvae recovered from the lungs in comparison to WT controls (Fig. 4A). Levels of IL-13 and IL-4 in the lungs of M3R^−/−^ mice were significantly decreased (Fig. 4B), and this was reflected by diminished numbers of IL-13^+^ and IL-4^+^ CD4 T cells (Fig. 4C), accompanied by reduced goblet cell hyperplasia and associated mucus production (Fig. 4D).

Immunity to *N. brasiliensis* can be accelerated by adoptive transfer of CD4 T cells sensitised by primary infection into naïve mice [20]. We used this approach to test whether optimal adaptive immunity is dependent on signalling through the M3R on CD4 T cells. CD4 T cells from previously infected WT and M3R^−/−^ mice were adoptively transferred i/v into naïve BALB/c mice and animals subsequently infected with *N. brasiliensis*. Recipients of WT CD4 T cells showed a significant reduction in the numbers of adult worms recovered from the small intestine at day 5 post infection, whereas recipients of M3R^−/−^ CD4 T cells showed no reduction in worm burden (Fig. 4E). This defective ability to reduce worm burden in recipients of M3R^−/−^ CD4 T cells was associated with an impaired induction of pulmonary alternatively activated macrophages (AAM) as demonstrated by reduced expression of RELMα and YM1 on alveolar macrophages (Fig. 4E). These data demonstrate that CD4 T cell responsiveness to cholinergic signalling via the M3R is essential for effective adaptive immunity to *N. brasiliensis*.

### M3R expression is required for CD4-mediated Th1 protective immunity to *S. typhimurium* infection

**Figure 5:**
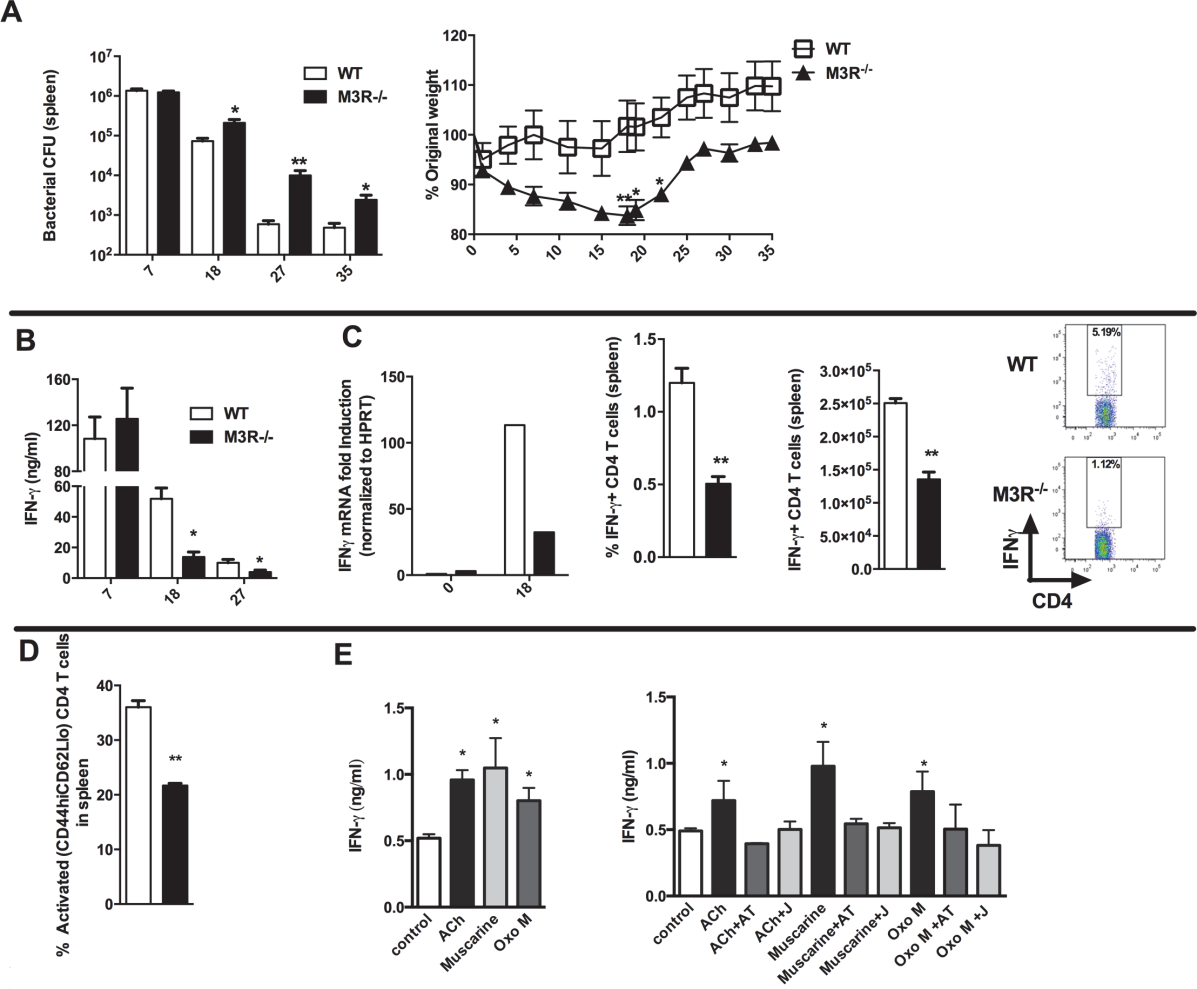
M3R deficient mice are more susceptible to *S. typhimurium* infection and exhibit impaired CD4 T cell responses. (A) Recovery of *S. typhimurium* from the spleens of infected mice at 7, 18, 27 and 35 days p.i. and weight loss at different days p.i. (B) IFN-γ secretion by antigen-restimulated splenocytes at different days p.i. (C) IFN-γ mRNA, proportion and number of IFN-γ+ CD4 splenic T cells assayed at day 18 p.i. (D) The proportion of activated CD4 T cells in spleens at day 18 p.i. (E) Secretion of IFN-γ by splenocytes stimulated with sub-optimal anti-CD3 after day 26 p.i. is enhanced by ACh and muscarinic agonists (10 μM). Enhancement of IFN-γ by ACh and muscarinic agonists is blocked by atropine (AT, 100 μM) and the M3R-selective antagonist J104129 (J, 40 nM). Data are shown as mean ± SEM, n = 5–6 and are representative of at least two independent experiments. Statistical significance was calculated using the Mann-Whitney two-tailed t test and denoted by *p<0.05, **p<0.01.

Having established that cholinergic signalling via the M3R contributed directly to CD4 Th2-driven immunity to nematode infection, we next tested whether this influenced immunity in a Th1 setting. We therefore infected WT and M3R^−/−^ mice with *Salmonella typhimurium*, control of which is dependent upon CD4 Th1 immune responses [16,17]. No difference in recovery of bacteria was observed early in infection (day 7), but at later time points M3R^−/−^ mice harboured significantly higher bacterial loads in the spleen (Fig. 5A) and displayed significant weight loss compared to WT mice (Fig. 5A). This increased susceptibility to infection correlated with reduced IFN-γ secretion by splenocytes at days 18 and 27 p.i. (Fig. 5B). Underlying this reduction in the protective cytokine response was reduced antigen specific IFN-γ production by CD4 T cells as demonstrated by both real-time PCR and flow cytometry analysis (Fig. 5C). Additionally, fewer activated (CD4^+^CD44^hi^CD62L^lo^) T cells were found in M3R^−/−^ mice in comparison to WT controls infected with *S. typhimurium* at day 18 p.i. (Fig. 5D).

### Cholinergic signalling through the M3R augments IFN-γ production ex vivo in splenocytes from *S. typhimurium* infected mice

To confirm that cholinergic signalling influenced T cell cytokine responses during *S. typhimurium* infection, we carried out *ex vivo* restimulation experiments with splenocytes removed at day 26 p.i. Both ACh and muscarinic agonists enhanced production of IFN-γ in T cells stimulated sub-maximally with anti-CD3. Moreover, as with ex vivo stimulation of T cells from nematode-infected mice, the stimulatory effects were blocked by addition of atropine or the M3R-selective inhibitor J104127 (Fig. 5E). Collectively, these data indicate that cholinergic signalling via the M3R plays a common role in enhancing CD4 T cell activation and cytokine responses in both Th1 and Th2 settings.

## Discussion

Understanding of the influence of cholinergic signalling on immune system function has thus far been largely restricted to innate immunity, principally focussed on dissection of the cholinergic anti-inflammatory pathway. The ultimate source of ACh in this reflex response is not neuronal, but a population of splenic noradrenaline-responsive CD4 T cells [21]. B cells also have the capacity of synthesizing and secreting ACh and, like T cells, this can regulate innate immunity, as B cell-derived ACh has been shown to inhibit neutrophil recruitment during sterile endotoxemia [22].

In contrast, little is known about cholinergic regulation of adaptive immunity, and the role of muscarinic receptors, despite numerous studies showing their expression on cells of the immune system [23]. A number of studies have observed that administration of muscarinic antagonists attenuates inflammation *in vivo*, suggesting a pro-inflammatory role for muscarinic receptors [24,25], although this conflicts with others which suggest that signalling through muscarinic receptors on vascular endothelium suppresses expression of cell adhesion molecules [22].

We demonstrate here that activation of the M3R is essential for optimal immune responses to two different pathogens; one which triggers Th2 cytokine production and another which triggers Th1 cytokines. In the case of primary infection with *N. brasiliensis*, absence of the M3R was associated with inhibition of smooth muscle contraction, reduced activation of CD4 T cells, lower IL-13 production and delayed expulsion of parasites from the small intestine. The impact of a lack of cholinergic signalling through the M3R was more evident during secondary infection, in which immunological memory in the lungs is particularly important [20] [26]. M3R deficient mice are highly susceptible to secondary infection, with a 4-fold higher recovery of parasites on day 2 p.i., lower levels of IL-4 and IL-13 in the lungs and reduced goblet cell hyperplasia in the airways. Mucus secretion by goblet cells is an important mediator of immunity to *N. brasiliensis* [27], and enhanced mucus production in the airways is correlated with reduced recovery of larvae following secondary infection [28]. Transfer of intact CD4 T cells from a prior infection confers protective pulmonary immunity to *N. brasiliensis* in naïve mice [20]. Using this approach we demonstrated that WT CD4 T cells from *N. brasiliensis*-infected mice were able to confer significant protection which was accompanied by expansion of AAMs in the lungs (which have recently been shown to be protective against lung stage *N. brasiliensis* infection [26]) of naive recipients, an effect which was not observed with CD4 T cells from M3R deficient mice.

The requirement of cholinergic signalling through the M3R for full CD4 T cell activation and cytokine production is not restricted to Th2 responses, but also extends to synthesis of IFN-γ and protection against bacterial infection. Our demonstration of an impaired response to the calcium ionophore ionomycin in M3R^−/−^ mice (Fig. 2) may well underlie this common M3R-dependent effect on Th1 and Th2 driven immunity, as sustained elevation of Ca^2+^ is necessary for the action of NFAT which in turn is required for full cytokine transcription in T cells [18]. Muscarinic receptor signalling has been shown to cause the rapid release of Ca^2+^ from intracellular stores and a sustained influx of external Ca^2+^ in neuronal cells [29] as well as T and B cell lines [23]. Moreover, the use of antagonists suggested that this was most likely acting through the M3R in lymphocytes [30]. Our demonstration that M3R^−/−^ CD4 T cells from *N. brasiliensis*-infected mice have an impaired ability to mobilize Ca^2+^ in response to ionomycin is indicative of a profound defect in cellular function.

No difference in recovery of *S. typhimurium* from the spleens of WT and M3R^−/−^ mice was observed early in infection (day 7), when control is largely affected through innate myeloid cells [31]. However, eventual resolution of infection is dependent on CD4 T cell-coordinated responses [32], and higher bacterial loads in M3R^−/−^ mice were associated with reduced IFN-γ from day 18 onwards (Fig. 5). Others have shown indirectly that cholinergic signalling can protect against bacterial infection. Thus, administration of paraoxon, a specific inhibitor of acetylcholinesterase, rendered mice more resistant to infection with *S. typhimurium* and enhanced production of IL-12. These protective effects were abrogated by co-administration of an oxime which reactivated acetylcholinesterase, implicating ACh as the agent driving potentiation of this response [33].

We show conclusively here that cholinergic signalling through the M3R is a major contributor to immunity to parasitic and bacterial infection, and that in its absence CD4 T cell responses are significantly impaired. Moreover, muscarinic agonists enhanced cytokine responses to both pathogens. The immunological defect in both scenarios may also be influenced by the lack of M3R expression on other cell types. However, the impaired immunity to *S. typhimurium* infection in M3R^−/−^ mice was not related to altered macrophage function and was also independent of M3R influence on B cells [34]. Our data therefore suggest that M3R-dependent protection against infection may primarily be a function of altered CD4 T cell responsiveness. CD4 T cells also express the muscarinic receptors M_1_, M_3_ and M_5_. However, our experiments with M3R-deficient mice and use of specific antagonists strongly support the conclusion that cholinergic enhancement of cytokine responses by CD4 T cells is driven by the M3R. This is reinforced by pharmacological experiments which indicate that elevation of intracellular calcium in T cell lines stimulated with muscarinic agonists is most likely effected through the M3R [30].

The current study represents an important development in our understanding of cholinergic signalling in immunity, and may have implications for the use of functionally selective M3R antagonists such as tiotropium, widely used clinically as bronchodilators to treat chronic obstructive pulmonary disease [35] and more recently asthma [36]. Several studies have shown that tiotropium can inhibit allergen-induced eosinophilia and cytokine production [25], while others conclude that the M3R promotes allergen-induced tissue remodelling and goblet cell hyperplasia without directly affecting inflammation [37], perhaps indicative of varying effects in different animal models. As expression of the M3R has been demonstrated on other immune cell populations including macrophages and dendritic cells (DCs) [23], these cells may also respond directly to ACh. Studies of human DCs have shown that cholinergic stimulation *in vitro* enhanced expression of receptors involved in antigen presentation (CD86 and HLA-DR) and influenced mixed lymphocyte reactions to these agonists. Moreover, these effects could be blocked by atropine but not mecamylamine, suggesting that signalling was effected via muscarinic rather than nicotinic receptors. However, cholinergic stimulation of LPS-treated DCs led to contrasting effects; namely a reduction in antigen presentation. These findings indicate that complex muscarinic-dependent effects on myeloid cells exist which may differ depending on, for example, the maturation status of cells [38]. Furthermore, a study on human DC from nasal mucosa showed that addition of metacholine to DCs upregulated Ox40L, which can induce and interact with Ox40 on activated T cells [39]. Both these data and our demonstration that ACh upregulates expression of Ox40 on T cells provide a mechanism for cholinergic enhancement of T cell activation and lymphoid-myeloid cell interactions.

In summary, our data indicate that signalling through the M3R promotes CD4 T cell activation and cytokine production in two disparate murine models of infection, suggesting that there may be potential for the development of drugs aimed at enhancing immune function for immunoprophylaxis or other applications.

## Materials and Methods

### Ethics statement

All animal work was approved by the UCT Health Sciences Animal Ethics Committee (Project licence 012/054) to be in accordance with guidelines laid down by the South African Bureau of Standards. Research at Imperial College was approved and in accordance with regulations of the Home Office (PPL70/6957).

### Mice

M3R^−/−^ mice were backcrossed to BALB/c background for 10 generations for this study. Mice were bred under specific pathogen-free conditions and used aged 6–8 weeks. Protocols for all experiments were reviewed and approved by the UCT and ICL Animal Ethics committees.

### Parasite and bacterial infection

For primary parasite infection, mice were infected subcutaneously with 500 *N. brasiliensis* infective larvae. To enumerate adult worms, mice were killed at various times post-infection (p.i.), intestines opened longitudinally, incubated in 10 ml saline for 3 hrs at 37°C and parasites counted under a dissecting microscope. For secondary infections, mice were treated at day 9 p.i. with ivermectin via drinking water to eliminate parasites, rested for 28 days and re-infected with 500 L3. Larvae were recovered from lungs by finely slicing the tissues, placing them in 5 ml saline for 3 hrs, and parasites subsequently enumerated.

*Salmonella enterica* serovar Typhimurium aroA^−^ (SL3261) is an attenuated strain of STm SL1344 (44) and was maintained and used to infect mice intraperitoneally with an infectious dose of 5 × 10^5^ CFU as described previously [40]. Tissue bacterial burdens were determined by direct culturing.

### In vitro CD4 T cell differentiation into Th1 or Th2 phenotype

CD4 T cells were isolated from mesenteric lymph nodes of naïve mice using flow cytometry (>99% purity). Sorted CD4 T cells were plated at 1 × 10^5^ cells/well on plates coated with 10 μg ml^−1^ anti-CD3 (BD Bioscience) and 5 μg ml^−1^ anti-CD28 (BD Bioscience). Th1 polarization conditions: 5 ng ml^−1^ rIL-12 (BD Bioscience) and 50 μg ml^−1^ anti-IL-4 (homemade Clone: 11B11); Th2 polarization: 50 ng ml^−1^ rIL-4 (BD Bioscience) and 50 μg ml^−1^ anti-IFN-γ. Cells were cultured in a final volume of 100 μl for 72 hrs, then transferred to fresh round-bottom 96 well plates and resuspended in appropriate antibody cocktails with the addition of 20 U ml^−1^ IL-2 (BD Bioscience) and cultured for another 48 hrs. Finally, cells were plated at 2 × 10^5^ cells/well and incubated in 96 well plates coated with 20 μg ml^−1^ anti-CD3 (BD Bioscience). Supernatants were harvested after 48 hrs restimulation and used for ELISA.

### Cytokine ELISA

Cytokine ELISAs were performed as previously described [41] using coating and biotinylated detection antibodies from R&D, with the exception of homemade coating antibodies for IL-4 (11B11) and IFN-γ (ANK18KL6). Streptavidin-conjugated HRP was used for detection with a commercially available substrate solution. MLN and lung cells were plated at 1 × 10^6^ cells per well in 48 well plates pre-coated with 20 ug ml^−1^ anti-CD3 and restimulated for 72 hrs. Homogenates of lung and intestinal sections were prepared using a Polytron homogenizer and all samples standardized to 5 mg ml^−1^ protein prior to ELISA.

### Calcium mobilisation assays

Changes in the cytosolic concentration of free Ca^2+^ were measured using the calcium indicator Fura-4-AM. Cellular suspensions of MLN were stained with anti-CD3 and anti-CD4 antibodies and then washed and resuspended to a concentration of 0.5 × 10^7^ cells/ml in Ca^2+^ flux buffer (Hank’s balanced salt solution (HBSS) containing 1 mM CaCl_2_, 1 mM MgCl_2_, and 0.1% BSA), and labelled with 5 μM Fura-4-AM for 30 min at 37°C in the dark. Labelled cells were washed in Ca^2+^ flux buffer. Changes in Ca^2+^ in CD3^+^CD4^+^ cells following stimulation with 10 μM ionomycin in the presence or absence of 10 μM EGTA were monitored by flow cytometry.

### Cholinergic stimulation

Cells were stimulated with 0.1 μg ml^−1^ (sub-optimal) anti-CD3, 10 μM ACh + 10 μM BW284C51, a specific inhibitor of acetylcholinesterases used to increase the half-life of acetylcholine (ACh), 10 μM Oxotremorine M (Oxo M), 10 μM muscarine or buffer controls for 48 hrs. The muscarinic receptor antagonist atropine (AT) was used at a concentration of 100 μM, and the M3R-selective antagonist J104129 [42] at a concentration of 40 nM. Supernatants were analysed for cytokines as described.

### Measurement of intestinal contraction

Jejunum sections (1 cm) were obtained and hooked onto a force transducer, placed in PBS maintained at 37°C in an organ bath, and stimulated with ACh from 10^−9^ M to 10^−3^ M. In between stimulations, the intestinal segment was allowed to return to baseline contraction (at least 5 min). All measurements were recorded using the Powerlab acquisition unit and analysed using the Chart5 program. The amplitude was measured as the difference between the peak and trough of the contraction and reported in millinewtons (mN).

### Flow cytometry

Single cell suspensions were prepared and 1 × 10^6^ cells incubated in PBS + 0.1% BSA, 1% normal rat serum and appropriate antibody cocktails. Cell populations were determined and acquired on a BD FACS Fortessa (Becton Dickinson). Cell populations were identified by the following antibody staining strategies: CD4 T cells: CD3^+^CD4^+^; CD8 T cells: CD3^+^CD8^+^; B cells: CD19^+^B220^+^; Macrophages: CD11b^+^F4/80^+^; Dendritic cells: CD11c^+^. CD4 T cells populations were additionally stratified into naïve (CD44^lo^CD62L^hi^) and activated (CD44^hi^CD62L^lo^) T cell populations and stained for Ox40 (CD134). Alternatively activated macrophages were characterised by staining for YM1 and RELMα. Intra-cellular cytokine staining was carried on MLN, spleen or lung cells. Cells were re-suspended in complete media (IMDM (GIBCO/Invitrogen; Carlsbad, CA), 10% FCS, P/S) at 2.5×10^7^/ml and stimulated with either10 **μ**g/ml PMA/ionomycin or antigen and GolgiStop (as per manufacturer’s protocol; BD Pharmingen) at 37°C for 4 hours. After re-stimulation, cells were surface stained for CD3, CD4 then fixed and permeabilized with Cytofix/

Cytoperm Plus (as per manufacturer’s instructions; BD Pharmingen). Intracellular staining was performed by staining cells with either IL-13, IFN-γ or appropriately labeled isotype control. All analyses were performed with FlowJo software.

### Adoptive transfer of CD4 T cells

CD4 T cells were purified from MLNs by positive selection using CD4 MACS beads (L3T4, MACS Miltenyi) according to the manufacturer’s protocol. Cells were further purified by flow cytometry to obtain purities above 95%, and 5 × 10^5^ purified CD4 T cells from infected or naive animals transferred into naive WT or M3R^−/−^ mice intravenously. Recipient mice were infected 24 hrs later with 500 L3 and killed 5 days post infection.

### cDNA synthesis and RT-PCR

RNA was extracted using the Qiagen RNeasy Mini kit as per manufacturer’s protocols. RNA was converted to cDNA using random primers and Superscript II. The following primer pairs were used M1R: 5’-GGACAACAACACCAGAGGAGA-3’; 5’-GAGGTCACTTTAGGGTAGGG-3’; M2R: 5’-TGAAAACACGGTTTCCACTTC-3’, 5’-GATGGAGGAGGCTTCTTTTTG-3’; M3R: 5’-TTTACATGCCTGTCACCATCA-3’, 5’-ACAGCCACCATACTTCCTCCT-3’; M4R: 5’-TGCCTCTGTCATGAACCTTCT-3’, 5’-TGGTTATCAGGCACTGTCCTC-3’; M5R: 5’-CTCTGCTGGCAGTACTTGGTC-3’, 5’-GTGAGCCGGTTTTCTCTTCTT-3’; β-actin: 5’-TGGAATCCTGTGGCATCCATGAAAC-3’, 5’-TAAAACGCAGCTCAGTAACAGTCCG-3’; IL-13: 5’-CTCCCTCTGACCCTTAAGGAG-3’; 5’-GAAGGGGCCGTGGCGAAACAG-3’

### Histology

Lung and intestinal sections were fixed with 4% formalin in PBS solution, embedded in wax and cut into sections, then stained with Periodic Acid Schiff (PAS) stain to distinguish mucus-producing goblet cells. The histological mucus index (HMI) was used to quantify pulmonary goblet cell hyperplasia in individual mice, as described before (49).

### Statistics

Values are expressed as mean ± standard deviation and significant differences were determined using either Mann-Whitney U test or ANOVA (GraphPad Prism4).
