# Correction: The M3 Muscarinic Receptor Is Required for Optimal Adaptive Immunity to Helminth and Bacterial Infection

**DOI:** 10.1371/journal.ppat.1004727

**Published:** 2015-03-19

**Authors:** The PLOS Pathogens Staff

The authors would like to correct the name of the fourth author, Fiona Culley. The correct name is Fiona Jane Culley.

The correct citation for this article is:

Darby M, Schnoeller C, Vira A, Culley FJ, Bobat S, Logan E, et al. (2015) The M3 Muscarinic Receptor Is Required for Optimal Adaptive Immunity to Helminth and Bacterial Infection. PLoS Pathog 11(1): e1004636. doi: 10.1371/journal.ppat.1004636
